# RUNNING, HEALTHY AGING, AND SOCIOCULTURAL CHANGE OVER 50 YEARS: PERSPECTIVES FROM OLDER ADULTS

**DOI:** 10.1093/geroni/igae098.3492

**Published:** 2024-12-31

**Authors:** Barbekka Hurtt, Seth Hudes, Shana Deters, Josie Allen, Asia Cutforth, Leslie Hasche

**Affiliations:** University of Denver, Denver, Colorado, United States; University of Denver, Denver, Colorado, United States; University of Denver, Denver, Colorado, United States; University of Denver, Denver, Colorado, United States; University of Denver, Denver, Colorado, United States; University of Denver, Denver, Colorado, United States

## Abstract

Running is a relatively easily accessible and perceived low barrier to entry sport for any age. This project goal was to understand how running is conceptualized as part of healthy aging over the past 50 years, given sociocultural and demographic changes that have occurred. Through recruitment on social media, with running groups, and at marathon events, participants completed survey and interview data on their life course experiences with running. Out of 37 survey participants, 5 were aged 61 or older with an average age 71 years old; of 21 interview participants, 5 were aged 61 or older. Survey results indicated that running was perceived as contributing to: a) positive increases in physical health attributes such as bone, cardiovascular, and immune health, muscle strength, and metabolism; and b) slightly or significantly more positive mood; conversely, all participants indicated their mood worsened when taking time off from running. Interview data supported the survey data and revealed positive perspectives of older athletes through phrases such as “motivating, strong, badass, happy, healthy, cool, active;” conversely, the majority of respondents felt “angry, upset, or frustrated” at the phrase “You play/run like an old person.” Slightly more than 50% of participants desired improvement for older adult community athletic opportunities, particularly those related to running. Yet, most older adult participants agreed that they “feel comfortable in my community to achieve my full athletic potential.” Community leaders, health, and aging services should consider opportunities to promote running groups for older adults that support both new and experienced runners.

